# Complexity and psychopathology: from mechanistic science to a physical understanding of the mental conditions based on chaos and complexity

**DOI:** 10.3389/fpsyt.2026.1764422

**Published:** 2026-02-23

**Authors:** Dimitri Marques Abramov

**Affiliations:** 1 Laboratório de Neurobiologia e Neurofisiologia Clínica, Instituto Nacional da Saúde da Mulher, da Criança e do Adolescente Fernandes Figueira, Fundação Oswaldo Cruz, Rio de Janeiro, RJ, Brazil; 2 Laboratório de Neurociências e Saúde Mental, Faculdade de Medicina de Petrópolis, Centro Universitário Arthur Sá Earp Neto (UNIFASE), Petropolis, Brazil

**Keywords:** complexity, mechanism, neurodivergence, non-extensive (Tsallis) statistics, psychopathology, reductionism, self-organization

## Abstract

The Traditional Scientific Method (TSM), rooted in the Cartesian-Mechanistic Paradigm, is insufficient for the comprehensive study of the human mind and suffering, which are complex phenomena. Thus, psychiatry can pathologize many vital critical processes and neurodivergences when it simplifies subjectivity and diversity through its nosological models. This essay advocates for a shift towards the Complexity Paradigm (CP)to achieve a psychopathology more adequate to human singularity. The CP posits that life and mind are emergent dissipative phenomena arising from the synergy between chaos and order, that enables systems to adapt through self-organization while maintaining their internal stability. Therefore, organic complexity appears to decrease with age and pathologies. We explore Non-Extensive Statistical Mechanics (*q*-Statistics, *qS*), proposed by Tsallis to describe neural complexity from EEG signal through the scalar parameter *q*. We’ve demonstrated that *qS*, by a *q*-exponential function, models the EEG non-linear behavior indicating its power for quantify the brain complexity, and also to describe modulation of the complexity by ageing or neurodivergence (attention deficit/hyperactivity condition in children) in the brain as a whole, and by different functional brain states (as in oddball attention paradigm or listening a preferred piece of music) in different brain regions. We hypothesize that neural complexity could be reduced in pathological mental conditions, but not in neurodivergence, neither in mental suffering that are critical processes of transformation. Assessing neural complexity as a property of the brain through *q*-statistics may be a method to a more comprehensive and precise psychiatry.

## Introduction

Five hundred years ago, the Traditional Scientific Method (TSM), formalized by Descartes, established a Mechanistic Science, stemming from Western Thought (1). The TSM, based on reductionist analysis, succeeded throughout human history. It took robots to Mars, cured deadly diseases, and manipulated quantum particles across space and time. Thus, Western Thought prevailed over all cardinal points, becoming the dominant paradigm even in the East.

Yet, under the bias of the TSM, Western Thought fails to answer emerging and relevant existential questions for human beings, such as “what is happiness?”. Questions born in the human mind, which unequivocally demonstrate critical processes experienced as suffering and manifested in the world, ranging from daily tensions to rupture with what is conceived as common reality.

Contemporary psychiatry is a medicine mandated to deal with these crises of the human mind when they gain clinical interest. However, psychiatry itself shows it is undergoing its own crisis: although the Mechanistic-Cartesian Paradigm has brought invaluable advances to medicine in general, it proves insufficient to capture the complexity of the human experience in suffering, as well as the tensions and ruptures with the world we inhabit (2–4).

It is reasonable to consider that Descartes’ Scientific Method was corrupted when applied to Psychiatry; the science he systematized presupposes a dualism between mind and body: *res cogitans* and *res extensa*, with empirical Science only finding relevance in the world of bodies (*res extensa*) (1). The philosopher knew the limitations of his method. However, starting from Positivist philosophy (5), the intangible nature of the mind was assimilated as an organic phenomenon on scientific investigation. And, according to Laplace, the mind simply was not yet decipherable due to our ignorance of hidden variables and the imprecision of measurements (6).

Once the TSM is, by the cartesian principle, insufficient to comprehend the universe of the human mind, the solution was to reduce the scientific perception of Life and Psyche to the limits of the TSM. And since then, in formal Western Thought, the complexity of the mind is invisible to TSM because Mechanistic-Cartesian Paradigm cannot apprehend what differentiates life and soul from the mechanical, the linear, and the objective. So, Western Thought established the clinical practice to a place that adopted mechanistic and reductionistic models for diseases. Indeed, at a high level of relevance, the TSM has provided invaluable advances in quality of life within the normality-disease dichotomy. Consequently, a place for human crises was established in dysfunctional organs in both formal science and the common sense. In this path, the medicine of the mind, Psychiatry, also adopted the Cartesian mechanism and positivist materialism as its epistemological paradigm under the view of the TSM.

However, Medicine, like other clinical disciplines, originated with Hippocrates as a science that holistically centers the person, and thus, the subtle and subjective elements also should be central in the medicine, placing it in the condition of an art (7, 8).

Human health of a human condition inhabited by crises and pain are much more than the normality-disease dichotomy. Yet, through the TSM, we cannot reach the complex dimension of Humanity.

However, We reiterate that the value of the TSM for the study of mental conditions is unquestionable. Nevertheless, its insufficiency is ignored, and the direct answers derived from it are simplified. The human complex, unequivocally critical by nature, cannot be reduced to mental disorders within the normality-disease dichotomy, as advocated by countless authors (3, 4, 9).

Despite nosological manuals (DSM and ICD) recommending some observance of human phenomena under cultural contexts, prevalent psychiatry ignores the protagonism of the social and cultural complex in determining mental conditions of clinical interest, however obvious the complex nature of these conditions may be (10, 11).

As we shall see, given the progressive inadequacy of reductionist mechanism and its STM to satisfactorily understand many dimensions of the world, the evolution of human thought has brought the Complexity Paradigm (CP), which has become the epistemological foundation for a new Philosophical Thought (12). We can begin to understand “complex” by looking at the possible etymology of this word: “that which is woven together” (12). The CP states that many (if not almost all) processes and structures in the Universe are systems whose components cannot be dissociated from one another, and can only be substantially understood as a whole. Given their essential integrality, the spontaneous microstates of their components combinatorially manifest an unpredictable variability of emergent macroscopic states, autopoiesis (13) by self-organization (14), and thus sufficient degrees of freedom to maximize the system’s adaptability. While the word mechanism refers to a “simple” system, the word “organism” is more appropriate for a complex system. The CP serves as a counterweight to the simplification, objectification, and mechanization of nature, life and psyche.

The CP brings several insights about the nature of processes and structures in the Universe that invites us to think differently, from Logics where “AND” becomes more plausible than “OR” to the decision-making processes of a CEO who observes his corporation from a systemic perspective: this is Complex Thought to cognitively operate on the CP.

From the CP also is born a new science that maybe can answer what life is and why it exists: a “science” of complexity (SC). For that, we have developed several non-reductionistic methods, that can empirically for example, qualitative research, crucial for the psychosocial sciences, which seeks consistent convergence through the analysis of the discourse of different people immersed in the same context, problem or theme (15). Statistical physics, through entropy analysis (16–19), and chaos physics (20–22) allow for the understanding of complex systems, whether physical, biological, social, or even economic (19).

Similarly, the CP with a SC can bring a better understanding of the mind, as well its divergent profiles and critical conditions, which are not simply disorders in themselves. Therefore, a new medical epistemology based on CP can be a path that would bring back the Hippocratic ideal for a person-centered medicine, sensitive to human diversity and its circumstances.

As a result, a SC is possible, but it may not take robots to Mars or produce psychotropic drugs. However, a Complex Science must substantiate more realistic perceptions that dignify the nature of the human singularity, providing new technologies that embrace the diversity, subjectivity, and “complexity” of this Universe and its possible horizons.

Advocating in favor of the necessity for a science centered on the CP to build a psychiatry worthy of humanity in its singularities and diversity is the purpose of this essay. We hypothesize a possible psychopathology based on CP where morbidities are not simply deviations from a physiological (or cultural) norm *per se*, but they can be understood as complexity loss, with reduction of degrees of freedom and subsequent maladaptive outcomes in the dynamics of a singular organism (individual as a whole) in relationship to a broader context, from biological interactions to the social universe. To this end, we will revisit the impasses of the TSM and extrapolate it into a synthetic and comprehensive description of the core foundations of Chaos and the Physics of Complexity for a more adequate scientific understanding of the human condition.

## The impasses of mechanistic science

In Traditional Western Thought, the TSM emerged beyond classical deductive thought: post-Socratic philosophies, especially Aristotelian and Cartesian ones, contributed immensely to the dawn of Galilean/Newtonian science and the development of scientific technologies. Both intellectual conquests of Humanity built a civilization capable of establishing democratic states and walking on other planets.

Since the physical and astronomical models from Copernicus to Newton, laws of Nature were unveiled, as if it were finally possible to hear the thought of God, as a pre-established universal order in a cosmos that works like a clock. The Enlightenment and Positivism marked the peak of the materialistic and reductionist view of everything (5). The almost absolute theoretical and practical precision regarding space and time was achieved. For four hundred years, the TSM was sufficient to meet society’s demands and anxieties. Until Maxwell’s equations, which unified magnetism and electricity, everything was perfect.

Flaws in the models were always noted when the TSM ventured outside physics and chemistry in controlled environments. In biology and physiology, phenomena did not appear so linear. And in medicine, the TSM was far from finding explanations for the human brain and mind.

Up to a certain time, the models’ failure and inaccuracy were treated as mere appearance, an effect on the surface of the clock, inherent to measurement techniques (observation error) or even the lack of sufficient knowledge to achieve model perfection. Since the 17th century, a mathematics of probabilities, statistics, formally emerged as a tool to compensate for our ignorance. The certainty, however, remained about a modelable mechanism underlying the data and the decks of cards. In this sense, the mathematician Laplace told us:

“An intelligence which, at a given instant, knew all the forces that animate nature, and all the positions of all the objects of which nature is composed, if that intelligence were ample enough to subject those data to analysis, it would encompass in a single formula the movements of the largest bodies in the universe and of the smallest atoms; nothing would be uncertain for it, and the future, just like the past, would be present before its eyes.” (6)

Laplace was wrong. With the evolution of mathematics and the discovery of non-linear dynamic systems, we began to demonstrate that the unpredictability is an inherent property of Nature (23).

And indeed, in the last years of the 19th century, Mechanistic Science began to encounter the insurmountable limits of the TSM, leaving not only human anxieties but also the understanding of the Universe as a clock, unsatisfied. One of the first insurmountable impasses of the mechanism advocated by Laplace is the emblematic Three-Body Problem, which revealed the unstable and unpredictable nature of deterministic systems when it demonstrated the impossibility of modeling the orbital dynamics of three bodies under the mutual effect of gravity (23).

Boltzmann revolutionized physics in the second half of the 19th century when he created Statistical Mechanics, which describes the dynamics of a gas in terms of statistics of molecular moments. Besides formalizing the relationship between the microscopic dynamics of the world and the macroscopic properties of this world, he did so by assuming the probabilistic nature of the Universe (24, 25). Heat flow and entropy were formulated based on a mathematics of probabilities:

(1) $${\text{S}}_{\text{BG}}=-k\sum p\ln p$$

Where the Boltzmann-Gibbs Entropy (*S_BG_*) is the negative of the sum of the probabilities *p* of the *W* possible states of a system of particles times the natural logarithm of those probabilities, times *k*, which is the Boltzmann constant (Equation 1). In a system with equiprobable states, ${\text{S}}_{\text{BG}}=k\ln W$. In the formalism of Statistical Mechanics, it is possible that a spontaneous heat flow can occur from the colder to the hotter place in a thermodynamic system. This does not happen (and likely never will) because this would be an infinitely improbable state of the molecular system. Similarly, a cracked cup could reintegrate, or the air in a room could collapse into a single point. Although these states are possible, according to mechanistic thought, they are inadmissible.

This small equation began to change the perception of the world. And, at the dawn of the 20th century, quantum mechanics, a counterintuitive and essentially probabilistic theory, showed that the Universe is also irrational. This irrationality was formally recognized through the Copenhagen Interpretation, which, in fact, does not satisfy mechanistic rationality (26).

Chaos probably had been imagined by Epicurus as Clinamen (27) evidenced by Poincaré, Boltzmann, and Quantum Mechanics. However, the concept of Chaos as a fundamental property of Nature was not yet formally present in Science. It is the foundation of new theories and a new scientific epistemology, with inspiring reflections in all disciplines of human thought: the Complexity Paradigm.

### Chaos

“When the atoms move straight down through the void by their own weight, they deflect a bit in space at a quite uncertain time and in uncertain places, just enough that you could say that their motion had changed. But if they were not in the habit of swerving, they would all fall straight down through the depths of the void, like drops of rain, and no collision would occur, nor would any blow be produced among the atoms. In that case, nature would never have produced anything.” (27)

Four hundred years before Christ, Epicurus may had intuited the existence of modern Chaos, which he called "Clinamen", while observing the non-linearity of the Nature. And, he perceived its creativity, which gave rise to the entire Universe.

Chaos is the emergent property of Nature that “determines” its inherent and primary unpredictability which could be allegorically compared to irrational numbers, such as π = 3.14159… which is the ratio between the measure of a circumference’s length and its diameter.

Laplace was partially correct, because, as π appears in this simple arithmetic, it is the result of an absolutely deterministic process. But as an irrational number, π has infinite, completely unpredictable decimal places, such as a chaotic behavior. To know progressively more of π, it is necessary to solve it numerically through the circumference/diameter arithmetic.

In line with Laplace, the Universe may indeed be determined by a set of mathematical functions, as Nature has infinite irrational numbers like π, and infinite non-linear functions like the differential function of the logistic map discovered by May in the 1970s (Equation 2) (28):

(2) x(t+1)=r·x(t)·[1−x(t)], for 1<r<4, and for x(1)<1.

Every atom in the Universe may be determined by several non-linear differential functions, which by no means implies predictability—or mechanism.

Through one of these functions, Edward Lorenz, a meteorologist and mathematician, conceptualized chaos in the 1960s upon the discovery of one of its most spectacular properties: the extreme sensitivity of chaotic dynamic systems to initial conditions (29). In a non-complex dynamic system, described by a system of linear functions like *y=ax*, the change in the value of a parameter *a* will produce a proportional deviation in the system’s evolution. In a complex system, small changes in the initial parameters, over time, produce progressively more unpredictable evolutions from the initial moment: the “Butterfly Effect.”

Chaos is an evident property at the microscale. In molecular systems, its expression is absolute, requiring a “statistical physics” to study these systems, as pioneered by Boltzmann and Gibbs: in the absence of certainties, we observe the possibilities and calculate the probabilities of the system’s evolution (24, 25).

The legacy of Boltzmann and Gibbs changed Science but was not enough to explain the relationship between Chaos and Nature. Why, at a macroscale, do the effects of Chaos seem to disappear in patterns such as tree leaves, quartz crystals, or human bones? From the probabilistic viewpoint of Boltzmann’s Statistical Mechanics, these are also infinitely improbable states. But, unlike heat flow against the current, these states appear everywhere.

### Complexity

From a physical perspective, Complex Systems are indissociable structures and processes whose components, though definable, maintain intricate connectivity through long-range correlations and direct interactions (19). However, their inherent holistic nature may not be sufficient for complexity, as information is also required. Information has a mathematical relationship with entropy (17), which in turn is related to system nonlinearities, primarily provided by chaos.

Without chaos and information, a hyperconnected Universe would likely resemble a supersymmetric crystal, where hydrogen molecules, stabilized by nuclear forces, interact with one another through gravity, forming a perfect network of equidistant and identical stellar bodies throughout the space-time fabric. On the other hand, if there were only chaos without either nuclear (short-range) or gravitational (long-range) connectivity, matter would likely form a diffuse and isotropic plasma cloud throughout the Universe, which could be perfectly modeled by the functions of Boltzmann-Gibbs statistical mechanics.

In this sense, without an optimal balance between order (connectivity) and chaos (unpredictability), complexity cannot emerge, and the system’s dynamics are neither creative, adaptive, nor evolutive and internally stable —nor healthy, as shall be understood in the following lines.

Neither of those imaginary universes are ours. Because from this chaos and connectivity, infinite patterns of order emerge in our Universe (*ordo ab chao*): the Earth’s orbit around the Sun, under the effect of gravity on an astronomical scale, as well as turbulence in the atmosphere forming a cyclone. These are manifestations of singular order that cannot be predicted by predetermined designs (or laws) or linear functions driven by fundamental forces. The leaves of a mango tree all follow a pattern; an order emerges from molecules and cells. But each leaf carries the evidence of Chaos in its singularity. Why do the untamed particles in one context manifest order in another context, when they are together giving shape to Nature?

These particles maintain connections between themselves, which can be local interactions, but also non-local interactions and correlations, like a series of falling dominoes, where the first has an absolute correlation with the last one. Or the effect of a neuron in the motor cortex on a muscle in the sole of the foot through a neuronal pathway and a neuromuscular synapse. In this sense, the force of gravity determines the connection between celestial bodies. These connections can form crystalline lattices, orbital systems, extremely simple if they are very strong. Conversely, where connections are inexpressive, “strong chaos” manifests, as in ideal gases.

Connections are the counterweight to Chaos, organizing systems where the components are restricted in their degrees of freedom, but still maintain enough freedom to collectively manifest, through self-organization, macroscopic states of order that locally reduce entropy. The mango leaf “emerges” through self-organization from a set of components naturally connected both at short and long distances.

We can understand the manifestation of Order from Chaos as the propagation of a non-linear perturbation in a region of the system that subsequently finds a state of entropic minimization (reduction of free energy)—an attractor: the complex system tends to self-organize into an energy-saving configuration.

Whirlpools and cyclones are a canonical image of attractors in chaotic systems, where energy dissipates, and the system finds stability. Other complex structures and organizations in Nature, like mango leaves or an enduring government, can be described as attractors when they dissipate energy, reduce entropy, and find stability. Both the driving machinery of a cellular flagellum with molecular rotors and stators (30), and the mental blueprint of the first mechanical turbine can be the manifestation of the same attractor in different contexts: one molecular, resulting from the self-organization of matter in the evolutionary process, and the other cognitive, resulting from the self-organization of neural activity in the process of mental representation. What many advocate as “intelligent design” might be the emergence of systematic and systemic patterns at the various scales and realities of a complex universe.

Thus, life, mind, and society are emergent phenomena that appear in the dance between chaos and order, through the intricate network formed in the intimacy of Nature’s structures and processes.

From the dynamics of chaos in complex systems, an adequate scientific definition for life, inspired by Ilya Prigogine’s work on dissipative systems, can be formulated (20, 21): life is an emergent dissipative phenomenon, the self-organization of a system complex enough to simultaneously manifest organization and unpredictability and evolve creatively and adaptively in the synergy between order (intrinsic connectivity) and chaos.

Accumulated evidence over decades demonstrates that pathological processes compromise the complexity of the organism (31–39). This perspective finds a masterful echo in the psychiatry of Henry Ey, who fundamentally defines mental disorder as a loss of freedom (40). In this theoretical framework, processes that uncontrollably increase chaos (like psychotic disorganization) and those that impose excessive and rigid order (like compulsive rituals) result in the same final consequence: the reduction of the system’s adaptive complexity (36).

It is possible that the compromise of complexity could scientifically describe a new psychopathology in suffering minds, allowing investigation into whether and how mental “diseases” exist. And if they do, what are they like? To this end, we need to physically describe complexity.

## The mechanism in complex nature

Mechanistic Science and its Cartesian method fail at a certain point because they ignore the Complexity Paradigm. They cannot answer the most complex questions because they treat nature as simple.

Mathematically, in the context of complexity, a multidimensional and diverse system with interconnected individuals cannot be known through reduction into its parts, because the network condition presupposes the sharing of information that makes the parts inseparable in relation to that system. To this end, I bring the allegory of the “hospital complex” where there is a subsystem that configures the surgical center. If moved to another institution, this surgical center will maintain some unchanged properties, but it will be a different surgical center, with different routines and different social behaviors among its workers. The Cartesian method cannot be satisfactorily applied where complexity is expressed.

The CP is blatant when we deal with deep artificial neural networks, which support Artificial Intelligence in Deep Learning (41): the more these neural networks are capable of learning, developing abilities, and manifesting intelligence, the less predictable, traceable, and interpretable the mechanism of that artificial intelligence is, compared to a “black box.” AI and its underlying NN, a work of engineering, return to the enigmatic context of the human mind and brain, where complexity cannot be accessed through the mechanistic and reductionist paradigm in the TSM.

From this, an agonizing question remains: how are we going to study the mechanisms of complex systems? The answer is not in surrender but in the recognition of a paradigm shift. We must accept that traditional mechanism offers an increasingly myopic view as complexity grows. The predictive models of complex systems will always be approximations. And, in this context, a mechanistic and reductionist medicine that aims to occupy itself transdimensionally with the bio-psycho-social context will not be satisfactory.

### Describing complexity

The American scientist Josiah Gibbs (25), who with Boltzmann discovered and formalized Statistical Mechanics, opened a parenthesis in the successful history of his physics of probabilities (which explains non-linear phenomena on a micro-scale in causal relation to the macroscopic states of systems): his equations could not explain all the probabilistic dynamics of Nature. They work well for ideal gases and analogous systems, but not for some other cases.

These “some other cases” are the vast majority of dynamic processes in the Universe. And, in fact, ideal gases and others under specific regimes of temperature and pressure (from atmospheric air to stellar hydrogen plasma) are the special cases. What is common are storms and ocean currents (turbulences), anthills and beehives, schools of fish and flocks of birds, distinct and individual molecules perfectly correlated to manifest orders in various dimensions, from the cell to the entire biosphere with epidemics and cities, air traffic, and the Internet.

Since correlations exist in complex systems, their components cannot be treated statistically as independent. The non-ergodic dynamics that characterize such systems cause the entropy manifested in them to evolve distinctly, diverging from traditional thermodynamic models, which generally assume an ergodic exploration of the state space (16, 19). What determines, in a few words, a different prediction about the Universe than what the first statistical physicists proposed?

Constantino Tsallis is a naturalized Brazilian who, almost 40 years ago, wrote the article Possible Generalization of Boltzmann-Gibbs Statistics (42), where he innovatively proposed a set of mathematical functions that would statistically model dynamic systems in general, including ideal gases, which are well-described by Boltzmann and Gibbs Statistical Mechanics. Complexity, on the other hand, becomes maximum when there is long-range correlation and interaction between the components of a system. There is a statistical dependence between the behavior of these components. Hence, the entropy of a complex system is defined by Tsallis (42) through the following entropic functional:

(3) $$S_{q}\equiv \frac{k}{q-1}\left(1-\sum_{i}p_{i}^{q}\right)$$

Where *q* is a non-extensivity parameter that describes the non-independence among the system’s components, determining consistent probabilities (Equation 3). That is, Tsallis proposed a theory that generalizes the Statistical Physics previously proposed: Non-Extensive Statistical Mechanics, or simply *q*-Statistics (16, 19). The core of NEMS lies in the consideration of the mutual interdependence among the system’s components and thus its complexity. This complexity is quantitatively related to a conditioning parameter, “*q*”. At minimum or negligible complexity, the value of *q* → 1. When *q > 1* in many models, we can infer a progressively more complex system as the value of *q* increases.

Over the years, experimental physics, engineering, sociology, economics, biology, etc., have shown the predictive power of *q*-Statistics for modeling phenomena such as quantum entanglement, tumor growth, critical cosmological systems (through delta-statistics), the evolution of the financial market, epidemics, and structure ruptures, among countless cases (e.g., 16, 19).

**Figure 1:**
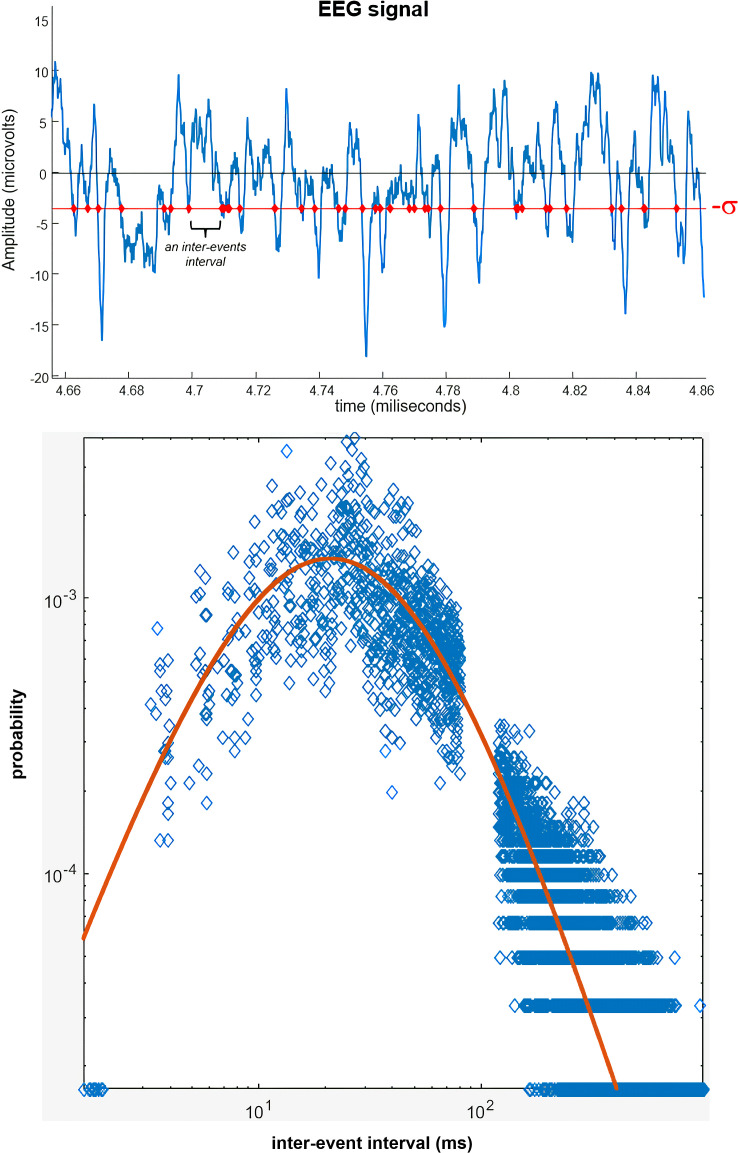
Analyzing the regularity of the EEG signal using q-statistics for quantifying neural complexity. Top panel: Extracting temporal distances between amplitude events that exceed -1 standard deviation from the mean of the negative part of the signal (red line with points on the events). Bottom panel: These distances are distributed into classes of a histogram, forming an empirical probability distribution (frequency of an interval class/total number of intervals computed). In this case, this distribution has 500 interval classes ranging from 0 to 1000 milliseconds and includes all intervals captured separately in the 20 EEG channels. The red line is the best fitting of the *q*-exponential function [Equation 4] by the computationally optimized least squares method. This example of best-fitting corresponds to the parameters *b* = 0.14, *c* = 2.24, *h* = 1.07, and *q* = 1.22. The parameter *a* is defined analytically from a gamma function with the parameters *b*, *c*, *h*, and *q*.

Our group has demonstrated the application of *q*-Statistics in modeling the dynamics of neural activity in the human brain on meso- and macroscopic scales, through EEG, which is notoriously complex (43). The regularity of the EEG signal, which represents the dynamics of possible brain states, is the parameter of analysis: we extracted the time intervals between events with amplitudes exceeding -1.00 standard deviation from the mean of the negative part of the signal (**Figure 1**, top). Instead of quantifying the entropy of the extracted inter-event interval series, we performed an empirical probability distribution of the occurrence of these intervals in histograms with 100, 500, and 1000 time interval classes (for example, 200 classes to intervals between 1 and 5 milliseconds, between 5.1 and 10 milliseconds, and so on). We fitted a *q*-exponential probability function developed by Tsallis (43) to the empirical distribution (**Figure 1**, bottom), and from best fitting, the function parameters are obtained, among them, the *q* value.

This function is given by:

(4) $$y_{q}=\frac{ax^{c}}{{\left(1+\left(q-1\right)bx^{h}\right)}^{1/\left(q-1\right)}},\text{ such that }\sum y=1$$

Where, in addition to *q*, the parameters *c*, *b*, and *h* must be determined numerically such that they best fit the function over a probability distribution of events of the system in question (43). These events are regularities of the EEG signal given by intervals between amplitude events less than -1 standard deviation of the signal. If EEG were a purely chaotic phenomenon, the distribution of the probability of occurrence of each interval should be modeled by the exponential function of traditional Boltzmann-Gibbs statistical mechanics:

(5) $$y_{\text{BG}}=\left(a_{\text{BG}}x^{c_{\text{BG}}}\right)/\exp\left(b_{\text{BG}}x^{h_{\text{BG}}}\right),\text{ such that }\sum y=1$$

But since EEG is a complex phenomenon, the function in Eq. 4 faithfully represents the probability distribution. In both eq. 4 and 5, the parameter a is analytically determined by a gamma function that predicts the total area under the curve equal to 1 – probability density function.

This *q*-exponential function that has described the complexity of the typical and neurodivergent brain with great sensitivity (44), demonstrating, for example, the phenomenon of reduction of neural complexity in EEG of all brain throughout natural aging, or the effect of different functional states (such as listening to a favorite song or performing an attentional task) on the complexity in specific brain sites (45).

In this sense, assuming that the complexity of a biological system decreases in pathological states (just as it decreases in aging), as several authors have demonstrated (33, 34, 46), could *q*-Statistics reveal whether a state of suffering or mental divergence is pathological, based on the measurement of the extracted parameter *q*?

Undoubtedly, from a mathematical perspective, the absolute values of *q* will depend to some extent on the experimental design, which defines the EEG signal resolution, the bounds of the function-fitting algorithms for empirical distributions, the resolution of these distributions, and so forth. Therefore, establishing the optimal configuration for any experiment and standardizing this setup remain the primary challenges for the universalization of this measurement, as it is inherently relative to this configuration of parameters. This is a milestone for a possible clinical validation and medical application of *q*-Statistics in the future, in parallel with the systematic observation of the relationship between the *q* parameter and the brain dynamics of typical, neurodivergent, critical (although still adaptive and healthy) and mentally disordered individuals, regarding their demographic profiles, in different cultures and societies.

The treatment of high-frequency (muscular) and low-frequency (eye movement) artifacts has not shown a significant influence on *q*-values concerning the adopted *bin* size and range for empirical distributions of inter-event intervals (45), although they should always remain a central concern for the interpretation of results. The choice of the threshold (-1 standard deviation) adopted in our studies is justified by the Central Limit Theorem from the perspective of *q*-statistics, in which the tails of Gaussian and *q*-Gaussian functions clearly diverge (19). Consequently, by establishing this threshold for events that exceed the central limit of the statistical model, the sensitivity for identifying complex dynamics may be increased (43).

### A psychopathology for the complex

In the physics of complexity, every system has critical regions or moments where/when disorder (entropy) increases, and the system must find a new attractor—a new configuration of states that re-stabilizes the dissipative system. Criticality can be spontaneous or result from interaction with other systems. In fact, it is criticality that allows the system’s adaptive evolution and the emergence of diversity (14, 47).

From the viewpoint of the Complexity Paradigm, the mental crisis can be a process of adaptive transformation of a system, while suffering is commonly the experience of the crisis, therefore a normal phenomenon of transformation and adaptation processes. Psychology historically recognizes crisis as a potential for adaptation, evolution, self-knowledge, and development of the psyche, and the emancipation of the individual acquiring their autonomy and self-determination passes through these processes (9, 48–50). From an empirical standpoint, studies correlate the adaptive stress response in healthy organisms with a transient increase in complexity (51–53). The allostatic response, which produces a deviation from the organic baseline, can logically correspond to subjective phenomena of suffering and discomfort. This is evident in common experience—for instance, in situations of physical discomfort under strenuous demand or psychic tension when solving everyday problems. Therefore, differentiating adaptive stress, which is consistent with a physiological allostatic response, from maladaptive stress, related to allostatic overload, remains a challenge in clinical practice, especially in psychiatry.

The perception of crisis as a pathological process that must be suppressed establishes a real risk for the individual’s evolution and, perhaps, for all of humanity. The suppression of crises is often achieved through drugs that modulate the stress response, such as anxiolytics or antidepressants (54, 55). These drugs are not curing diseases, but rather anesthetizing human experiences that should be worked through based on their subjectivity (56, 57).

The human individual is a singularity that extends beyond their material limits (49). The manifestation of new states of order (new attractors) stemming from a crisis occurs in longitudinal correlation (historical and current) with the world the individual lives in (50). In fact, our subjectivity is a complex that involves the world that interacts with and correlates to us. Therefore, like any complex, we are an inseparable part of a larger world, just as we are an inseparable collectivity of smaller parts. And so, successively, in both directions. We are part of a universal fractal.

Thus, the unfolding of the crisis must pass through these two dimensions: the microscopic (internal and biological) and the macroscopic (ecological, social, and cultural). In the 17th century, Spinoza wrote an “Ethics” that centers the role of affect (power to affect and be affected), that is, connections and correlations, where bodies indeed become a circumstance of their relations (58). Spinoza’s Ethics was a disruptive view in its time, contemporary to the Cartesian TSM, about the nature of things, including a God “emerging” from all bodies in “affection.” Spinoza was a philosopher of complexity, of a fundamental and paradigmatic philosophy that can unfold into a dynamic and equally complex mental health practice, to which the philosopher Paulo de Tarso Peixoto contributes, developing a “Physiology of Contact” and a “Psychopathology of Complexity” (49). Just as the integral of the atom comprises the Infinite (59), we are an inseparable part of an integral Universe, distinguishable from the whole like a wrinkle in a sheet, where unpredictable phenomena are also the primary cause of Nature’s dynamics.

From the viewpoint of Positivism, the classic Enlightenment scientific philosophical doctrine that guides biomedical rationality to this day, the CP is not recognized. Its mechanistic models, instrumentalized by the TSM, are still the biomedical objective in psychiatry, however much failure imposes itself. Thus, an initial epistemological error was denounced by the philosopher Canguilhem (1966): the fragility of the dichotomous binomial normality-disease, establishing him as one of the most important thinkers for human health, with special interest in mental health.

Like Spinoza, Canguilhem reveals a second philosophy of Complexity when he questions that pathologies and normality are defined based on normative parameters already established in physiology and, by corollary, also in social and moral norms, proposing, alternatively, that normality in the clinical universe is related to an organism’s capacity to oppose established norms (60). Discussing from this point, normality is related not to crisis, but to the individual’s potential to adapt and evolve from the crisis, finding a new attractor through emergence—autopoiesis.

The capacity to oppose already established norms (including physical ones) reveals the potential for adaptation and creative innovation that a complex individual is capable of developing within a biopsychosocial context. Not so rarely do we observe people who existentially begin to live after the diagnosis of intractable cancer. Normality must transcend the mechanical and stationary perception of life. According to positivist normality, evolution and diversity would be impossible. Every evolutionary leap begins with a born anomaly, which experiences tension (sofrimento) in the current state and seeks (and finds) a new contextualized state, where it thrives divergently and amplifies the potencies of humanity as a whole.

Under the epistemological and methodological limits of the TSM that impose its blatant insufficiency, psychiatry adopted a new nosology under a phenomenological rationality, systematizing the recognition of mental syndromes, which are classified in the DSM and ICD diagnostic manuals. As already mentioned here, this new nosology moved away from the once unshakable mechanistic and reductionist view when it contextualizes phenomena, for example, within culture—formally, the report of hearing voices from beyond in one cultural and social context is hallucination, in another, it is mediumship (61, 62).

However, the classification of a syndrome of clinical interest—or a Mental Disorder—is based on the assessment that it causes significant suffering and impairment across multiple life contexts. Such a conclusive perception must be grounded in a literally complex clinical act, involving an understanding of the individual through intersubjective interaction between the clinician and the subject, which typically requires a significant amount of time. Even then, the dichotomous categorization of Health versus Disorder can be a trap; at times, it is impossible to determine with certainty whether a crisis is adaptive or maladaptive, or whether it is endogenously triggered or—as presupposed by Complex Thought—is a phenomenon shaped by hegemonic cultural values and social constraints acting upon singular endogenotypes. In a snapshot, an individual may violently suffer when losing a job or facing the prospect of war; however, within their longitudinal existence (past and future), there lies the potential for reinvention, opposition to norms, and reorganization into a new attractor.

As a mere illustration, we bring up that in any discipline of innovation (and entrepreneurship), there is a consensus that every technological solution comes from the anguish of experiencing a problem (63). Surely, innovation, whether in the subjective/existential territory or dependent on problems and crises. However, not every crisis, in fact, will unfold into innovation. It is probable (and often, highly probable) that a critical point will lead the system to absolute disorder, maximum entropy—what we would call death.

The challenge of Psychiatry is to find a way to characterize the meaning of the suffering and the multidimensional impairments that the individual experiences in the occurrence of a syndrome to design the most appropriate intervention: from guidance and counseling to pharmacological and hospital intervention.

At this moment, we find in the physics of Tsallis’ Complex Systems an opportunity to develop a feasible solution that assists the clinician in their conduct and contributes to the understanding of the crisis being observed.

### Complexity through *q*-statistics and mental health

Over the decades, with the advancement of complexity science, numerous methods have emerged based on information-theoretic analysis (e.g., the traditional Lempel-Ziv method for Kolmogorov complexity, see 64) and chaos quantification (especially through the calculation of the Lyapunov exponent in nonlinear series, see 22, 65). In this scenario, entropy measures such as Sample and Approximate entropies (66), as well multiscale strategy for entropy estimation (18), are pivotal for making inferences regarding a system’s complexity. However, these commonly adopted methods rely on Shannon/Boltzmann-Gibbs entropic functions. Although they demonstrate adequate performance in many contexts, they fail to account for the non-additive nature of complex systems, which are inherently non-extensive. Consequently, classical formulations of entropy remain insensitive to the fundamental properties that define a system as truly complex.

Tsallis’ Complexity has been treated as a physical measure that synthesizes the complexity of a dynamic system (16, 19), such as the human brain (14). Since pathological states generally seem to reduce the complexity of a system, just as aging does, might it be possible for disadaptive (pathological) conditions of the mind to be synthetically inferred by the reduction of the complexity of the cerebral dynamics recorded in the EEG? This inference is synthetic and comprehensive, because Complexity is a property, not a physical force or mechanism.

This is our current scientific question, still unanswered: how does mental suffering interfere (if it interferes) with the Complexity estimated by the parameter *q* in the EEG in various different functional states? The premise being that the reduction of complexity (evidenced by the value of parameter *q*) correlates with disadaptive critical processes that would more likely lead the system to collapse rather than to reorganization through the stabilization of a new attractor.

Interestingly, we find, in line with other studies (67, 68) that neural complexity seems to be higher in children diagnosed with ADHD (44). And unpublished data from adults with an inattentive/hyperactive profile denoted through screening by the Adult ADHD Self Report Scale seem to corroborate this finding. However, the studied adults in this sample do not manifest impairment or suffering related to the inattentive and hyperactive characteristics. The specific meaning of this finding is not the object of this essay. Although, based on the criticisms pointed out against conventional psychiatry and in the light of Canguilhem, we may be demonstrating a potentially adaptive neurodivergent pattern that gained a pathological character due to the positivist bias of a nosology based on pre-established norms. Despite its resonance with other studies, these preliminary evidences regarding the sensitivity of *q*-Statistics in discriminating different mental profiles and states must be systematically validated through further research. Future studies employing diverse approaches are necessary to ensure with certainty that unforeseen extrinsic biases are not responsible for these findings.

Currently, we are collecting EEG data from adults and children with various mental conditions (Schizophrenia, PTSD, disadaptive disorder, autism spectra) and hope soon to be able to verify how neural complexity behaves in these populations, envisioning the possibility of understanding critical human and neurodivergent conditions, corroborating a psychopathology of complexity (49) and, perhaps, establishing a method for clinical assistance.

## The measure of complexity and the positivist dichotomy of normality and pathology

The proposal of this scientific and quantitative strategy to describe a psychopathology, contrary to what it may seem, does not circularly end in the positivist paradigm of normality-pathology, criticized by Canguilhem. Firstly, because we are not concerned with analyzing a neural tangle, extrapolating findings to infer the mechanisms of the mind, which are as inaccessible by the TSM in the human brain as in artificial neural networks in deep learning. Much less with these mechanistic models of normality, to classify people based on their individual deviations from the established norm in the model.

We are concerned with establishing an understanding of the individual’s capacity to build new norms for themselves through the measurement of complexity by *q*-Statistics, perhaps a measure of precision. In this context, *q*-Statistics is one more look at the person, contributing to Hippocratic clinical practice. Because we are certain that the value of *q* is the result of endogenous biological circumstances, but also ecological, social, and cultural ones in this inseparable holos, these dynamics.

**Figure 2:**
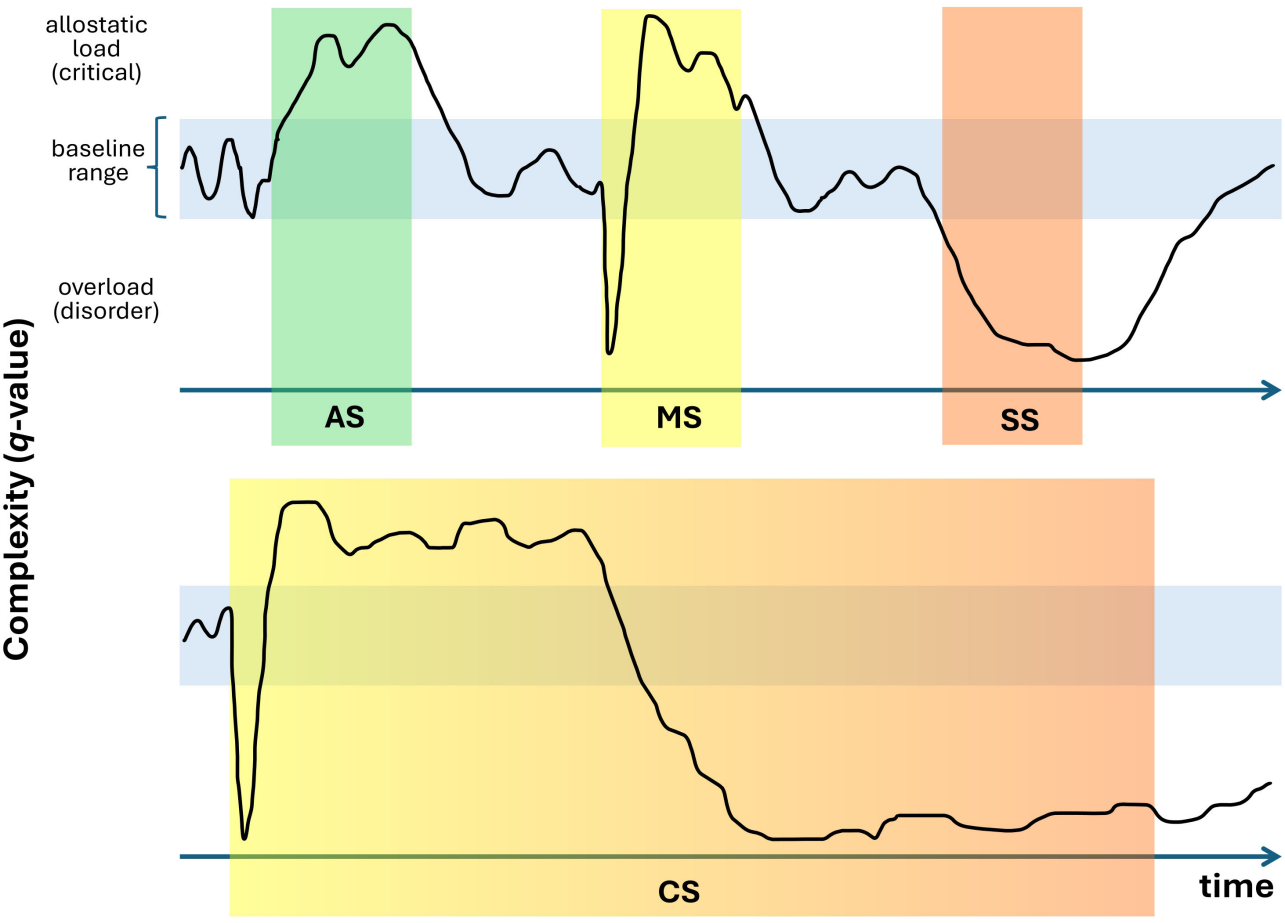
Speculative (non empirical) model regarding q-value dynamics across various mental contexts and their possible utility for clinical psychopathology. Based on the premise that the *q*-value—obtained by fitting a *q*-exponential function to the empirical probability distribution of time intervals between ongoing EEG amplitude events—faithfully reflects brain complexity, this metric is expected to fluctuate over time. These fluctuations occur between specific thresholds (either individualized or population-based) in association with real-world stressors (e.g., exposure to violence), phantasmatic/internal stressors (e.g., activation of deleterious self-beliefs, financial worries), or endogenous stressors (e.g., activation of genetic cascades triggering brain dysfunction). Top panel: The model assumes subtle increases in the q-value during adaptive everyday stress (AS) such as cognitive efforts to problems solving, a fluctuation of complexity with subtle reduction and subsequent compensatory adaptation in mild stress (MS), and sustained complexity loss during severe stress (SS), consistent with an established mental disorder. Bottom panel: The transition from a critical adaptive state to a clinical disorder may occur following any acute onset stress that becomes severely maladaptive through chronicity (CS). These hypothetic models follow Selye’s General Adaptation Syndrome (69) and may be quantitatively described through *q*-value dynamics.

As other complexity approaches (e.g. 53), and observations regarding *q*-values from EEG in different brain functional states (45), the value of *q*, while synthesizing the state of complexity, may vary at any instant, from calmness, crisis and mental disorders, among different thresholds (**Figure 2**), implementing a supplementary clinical assessment strategy that reliably supports decision-making in the field of psychiatry.

It is possible that we can empirically establish a narrow and specific range of *q*-values ​​for the brain complexity of healthy and typical individuals and those with different mental disorders and conditions. After all, the standard deviation of the q-values ​​we obtained was on the order of 5%, in an age range that varied between 21 and 87 years (45). After analytical solution of the *b* and *h* parameters through functions estimated by linear regression, we found a highly accurate (*c* x *q*) map that classified samples of typical children and those with ADHD into completely segregated clusters (44). Further studies have been achieved by our group to explore and confirm these first findings.

However, the dynamics of *q* for this individual may qualitatively denote their evolution toward strong chaos or toward a new possible order, in light of their context. Just like the expression of affect, the flow of thought, and the fluctuations of attention, the value of *q* is one more psychopathological dimension, extremely sensitive to mental criticality, rather than a biomarker for a specific diagnosis.

In this sense, although the value of *q* appears to synthesize complexity as a fundamental property—one increasingly associated with states of health and pathology—the potential clinical utility of q-statistics will always fall short of the clinical act itself. As this essay has consistently advocated, we reiterate that the clinical act is, and should always remain, a comprehensive, interpersonal, and multidimensional exercise—one that is attentive to history, individual and collective values and beliefs, and also to psychopathological indicators – among them, possibly quantitative description of the absolute or relative current complexity.

Thus, there should be no doubt that a complexity-based psychiatry is not limited to physical markers of complexity (which would be a paradox in light of CP), just as it cannot be reduced to a practice of diagnostic classification and protocol-based intervention. It must express an awareness of the legitimacy of human diversity in all its circumstances, focusing primarily not on diagnoses or classifications—which can objectify, simplify, and reduce the human being, as well as distort medicine from its humanitarian principles. Psychiatry, like any medical field, must primarily concern itself with the autonomy and emancipation of the individual, as well as with equity among people.

## Final considerations

Constantino Tsallis serenely admits not being able to define “complexity” in words. He teaches us that it is easier to experience it, like “beauty”: we recognize it through what we feel, but we cannot define it objectively. We also know love and all other human emotions not by concept or descriptions, but by experiencing them (70).

Beauty can be recognized as the valuation of a pattern of shapes and symmetries, and love correlated with significant events with behavioral and physiological developments (71). A scientific way of thinking teaches us about beauty and love, but they are not sufficient to bring us full understanding, falling short of the subjective and collective experience. In this sense, in a psychopathology of complexity (49), the emotions that beauty evokes are manifestations of a complex and singular context that transcends the delimitations of the individual material body, just as it transcends the delimitations of a descriptive conceptual definition by verbal or mathematical language.

Tsallis’ reflection and the allegory of the irrationality of emotions induce us to perceive the nature of complex systems, justifying the necessary care in the pretensions of knowing the world through the TSM, abandoning all other wisdom and experiences about the Universe, at the risk of our simplification and dehumanization. The criticism of the application of scientific reductionism to mental health is also clear, at the risk of simplifying humanities, mechanizing people, collapsing individual identities, eliminating diversity, and paralyzing (if not degenerating) the evolution of humanity.

According to Kuhn (72), science does not advance linearly and cumulatively but through crises that produce disruptions for new directions. At that moment, a new paradigm emerges, offering a new theoretical and methodological basis satisfactory for unresolved problems and attracting a new generation of scientists. Everything indicates that science is at an epistemological critical point since the beginning of the 20th century, because the Mechanistic Paradigm and its Method no longer satisfy the evolution of this science, demanding a new paradigm, which is Complex Thought. And this crisis may be deleteriously unfolding through contemporary psychiatry upon subjective existence.

Some may understand this discourse as “antipsychiatry” due to the pungent criticisms. On the contrary, this discourse is the call for a complex psychiatry centered on the singularity of the person and their life, which gathers philosophy, art, creativity, sensitivity, and empathy in the search for the best well-being for every suffering person, and armed with valuable cartesian science, but one that was never sufficient nor is, nor should be, the axis of psychiatric thought/feeling. Building upon an arguably overly rigorous epistemological critique of psychiatry (4), this work has sought to evolve towards concrete proposals that can enrich contemporary psychiatric practice. While we fully acknowledge the invaluable advances brought by the TSM.

We conclude that an adequate scientific psychiatry, which should go beyond the traditional mechanistic and reductionist rationality, establishing itself as a basis for a clinical practice based on the understanding of individual potentialities and, distinctly, the processes of crisis, adaptation, and mental illness concerning typical and neurodivergent people, is only possible through the Paradigm of Complexity and their scientific endowments and tools.
